# Initial insights into the relationship between symptomatic COVID-19 disease and specific clinical and blood parameters in patients with alcohol use disorder

**DOI:** 10.3389/fpubh.2026.1773558

**Published:** 2026-04-23

**Authors:** Lea Kozina, Divna Blažev, Danijela Zakić-Milas, Zrnka Kovačić Petrović

**Affiliations:** 1 University Psychiatric Hospital Vrapče, Zagreb, Croatia; 2 The Institute of Social Sciences Ivo Pilar, Zagreb, Croatia; 3 School of Medicine, University of Zagreb, Zagreb, Croatia

**Keywords:** alcohol craving, alcohol use disorder, blood parameters, cognition, COVID-19

## Abstract

**Background:**

Patients with alcohol use disorder (AUD) are at higher risk for long-term Coronavirus Disease 2019 (COVID-19) related consequences, due to bidirectional relationship and shared biological pathways between chronic alcohol use and COVID-19 disease. However, they are significantly underrepresented in the literature examining adverse outcomes associated with COVID-19 disease.

**Objective:**

Therefore, we comprehensively examined the initial group differences between AUD patients who experienced symptomatic COVID-19 disease and AUD patients without a history of COVID-19 disease. We investigated differences between COVID-19 and non-COVID-19 groups in AUD severity (alcohol use, alcohol craving), cognitive functions (assessed by the Montreal Cognitive Assessment), and basic blood parameters.

**Methods:**

This pilot study was conducted in two study points 6 months apart. Participants were 32 COVID-19 AUD patients and 31 non-COVID-19 AUD patients, who met rigorous inclusion and exclusion criteria. Sociodemographic questionnaire, COVID-19 interview and validated measurement instruments (5.0.0. Mini International Neuropsychiatric Interview, M.I.N.I.; Penn Alcohol Craving Scale, PACS; Alcohol Use Disorders Identification Test, AUDIT; Montreal Cognitive Assessment, MoCA; Clinical Institute Withdrawal Assessment for Alcohol Scale, Revised, CIWA-Ar) were assessed. Also, blood parameters were measured: erythrocyte sedimentation rate (ESR) and C-reactive protein (CRP) as inflammatory; alanine transaminase (ALT), aspartate transaminase (AST) and gamma-glutamyl transferase (GGT) as biochemical; total cholesterol (TC), low-density lipoprotein (LDL), high-density lipoprotein (HDL), and triglycerides (TG) as lipid parameters.

**Results:**

In the COVID-19 group, 93.7 % of participants had a mild form of the disease. There was significant difference between the two groups in cognitive functions which improved in the non-COVID-19 group, whereas they remained unchanged in the COVID-19 group [*F*_(1, 56.157)_ = 6.875, *p* = 0.011]. TC showed distinct pattern over time in the COVID-19 group [*F*_(1, 49.997)_ = 18.234, *p* = < 0.001]. CRP showed a weak negative relationship with the time since recovery from COVID-19 (*b* = −0.025, *p* = 0.009). The non-relapse group also showed an improvement in cognitive functions after six months, in contrast to the relapse group [*F*_(1, 56.157)_ = 6.879, *p* = 0.011]. In all participants, HDL levels increased after six months [*F*_(1, 58.036)_ = 4.604, *p* = 0.036].

**Conclusions:**

Cognitive functions and blood parameters require long-term follow-up in COVID-19 AUD patients. Better clinical and blood outcomes in non-relapse group support the overall benefits of reduction in alcohol use.

## 1 Introduction

There is a bidirectional relationship between chronic alcohol use and the Coronavirus Disease 2019 (COVID-19). Individuals with alcohol use disorder (AUD) are at increased risk for the Severe Acute Respiratory Syndrome Coronavirus-2 (SARS-CoV-2) infection, complications of COVID-19 disease, hospitalization and mortality (1, 2). The reasons for these are: multiple somatic and psychiatric comorbidities, impaired immunity, increased susceptibility to viral infections, reduced lung function, reduction of the antioxidant glutathione in the lungs, and an increased risk of developing acute respiratory distress syndrome (ARDS) (3, 4). The likelihood of these adverse COVID-19 outcomes increases with greater AUD severity (5). Vice versa, individuals who experienced COVID-19 disease are at increased risk of developing AUD. Furthermore, problematic alcohol use is a potential manifestation of long COVID (6).

Chronic alcohol use and COVID-19 disease affect and damage the same organ systems (4, 7). SARS-CoV-2 can cross the blood-brain barrier and potentially cause cognitive impairment through different mechanisms: neuroinflammation, viral encephalitis, hypoxia, cerebrovascular incidents, and neurodegeneration (8). A particularly vulnerable group for cognitive decline after COVID-19 disease are AUD patients, as alcohol use negatively impacts cognitive functions due to direct toxic effects and the numerous alcohol-associated somatic comorbidities, which potentially lead to dementia (9). Furthermore, recovered COVID-19 individuals may have significant abnormalities in basic blood parameters (4, 10–12). An increase in C-reactive protein (CRP) and erythrocyte sedimentation rate (ESR) have been observed in individuals reporting long COVID symptoms (10). Liver dysfunction has been observed in up to 50 % of patients with acute disease and elevated levels of alanine transaminase (ALT), aspartate transaminase (AST), and gamma-glutamyl transferase (GGT) may persist long after acute COVID-19 disease (11). Studies show impaired levels of low-density lipoprotein (LDL) and high-density lipoprotein (HDL) in hospitalized patients upon admission, with improvements in lipid levels not occurring until 6 months after recovery (12).

Likewise, chronic alcohol use is associated with impairments in inflammatory, biochemical and lipid parameters (4, 13). Alcohol use induces gut leakage, intestinal inflammation with increased entry of pathogens into the blood, thus promoting systemic inflammation with elevated inflammatory markers (3, 4, 7). Furthermore, alcohol has been proven to alter HDL, LDL, triglycerides (TG) and total cholesterol (TC) levels in the blood (13). Alcohol use negatively affects hepatic fibrosis signaling pathway and it can cause various forms of alcohol-related liver disease even in small amounts (4).

Only a small number of studies have examined the acute consequences of COVID-19 disease, while also including patients with AUD (7, 14). A retrospective study reported that patients with a history of alcohol abuse had a higher likelihood of experiencing dyspnea and at least one neuropsychiatric symptom during acute COVID-19 (14). The authors suggest implementing routine alcohol abuse screening in patients presenting with symptoms suggestive of COVID-19 (14). Furthermore, AUD and COVID-19 disease jointly contribute to psychological (through cognitive and emotional dysregulation) and biological (through gut dysbiosis) changes, which could represent a mechanism behind the development of neuropsychiatric symptoms, even long after recovery from acute COVID-19 disease (7). The severity of AUD also appears to have intensified during the pandemic, particularly among individuals with pre-existing mental health conditions (7, 15). The number of delirium tremens episodes and the prevalence of psychiatric comorbidities increased in the population of AUD patients (15). Patients with AUD are more susceptible to pandemic-related stressors compared with the general population, and these stressors constitute a substantial psychosocial risk for relapse (16, 17). Thus, chronic alcohol use and COVID-19 disease may exert an amplified impact through bidirectional relationship and shared pathophysiological pathways in AUD patients with a history of symptomatic COVID-19 disease, possibly leading to long-term consequences (4, 6, 7). However, comprehensive investigations of this interaction are still lacking (6, 7).

For these reasons, we aimed to comprehensively examine the initial group differences between AUD patients who experienced symptomatic COVID-19 (exposed/COVID-19 group) and those without a history of symptomatic disease (non-exposed/non-COVID-19 group). We assessed AUD severity (alcohol use and craving), cognitive functioning, and blood parameters (ESR, CRP, GGT, ALT, AST, TG, TC, LDL, HDL) at two study point six months apart. The aim of this pilot study is exploratory in nature, with its primary purpose being to lay the groundwork for a larger, comprehensive research project. We expected that the COVID-19 group would exhibit more severe AUD symptoms, poorer cognitive performance, and greater disturbances in blood parameters (more pronounced liver impairment, higher inflammatory markers, and hyperlipidemia) in the long-term.

## 2 Methods

### 2.1 Study design

This pilot study was conducted in two study points (1st from March 2023 to March 2024 and 2nd 6 months after). The study is a part of the larger prospective observational project examining the association of symptomatic COVID-19 disease with psychiatric symptoms in AUD patients, which was conducted from March 20, 2023, to March 21, 2025 (follow up is still going).

In this study 32 COVID-19 AUD patients and 32 non-COVID-19 AUD patients were recruited consecutively during AUD treatment (hospital, daily hospital or outpatient) in the Department of Addiction at University Psychiatric Hospital Vrapče in Zagreb, Croatia. The first assessment was at least 10 days after the establishment of initial abstinence (in order to reduce acute effects of alcohol intoxication and withdrawal) and the second assessment was six months later. Since the most severe alcohol withdrawal symptoms typically occur within the first four days after cessation of alcohol use, we considered a period of 10 or more days sufficient for the acute effects of alcohol to diminish (18–20).

Conducted G^*^Power analysis suggested that this sample size, for repeated measures, within-between interaction design, and assumed correlation of 0.5 between measurements, can be sufficient to detect effect size of *f* = 0.18, or small to moderate effects.

Some participants were not able to maintain abstinence until the second evaluation, and the impact of relapse on the severity of symptoms and blood parameters was statistically controlled by including relapse as a moderator in all analyses.

The Ethics Committee of the University Psychiatric Hospital Vrapče, Zagreb, Croatia in written form approved the study in accordance with the Declaration of Helsinki standards.

### 2.2 Participants

A total of 64 participants included in the study provided informed consent following the ethical principles in human research.

The diagnosis of AUD for all participants was determined according to the International Classification of Diseases, 10th revision (ICD-10) (21), the Diagnostic and Statistical Manual of Mental Disorders, Fifth Edition, Text Revision (DSM-5-TR) (22) and the 5.0.0. Mini International Neuropsychiatric Interview (M.I.N.I.) (23). Our participants met the criteria for alcohol dependence according to ICD-10 (21), for alcohol use disorder according to DSM-5 (22), and for current alcohol dependence according to the M.I.N.I. (23). In Croatia, ICD-10 is still the official diagnostic classification system, not ICD-11, which is why it was used in this study. The same diagnostic framework was applied in our previous study (24).

Participation was voluntary and excluded financial or any other compensation.

The inclusion criteria were:

(1) treatment-seeking AUD patients who have not abstained from alcohol for the last 12 months during the first assessment,
(2) negative alcohol test in exhaled air and negative urine test for illegal psychoactive substances on the assessment day,
(3) usual treatment for AUD, including pharmacotherapy (anxiolytics, medications to treat AUD, hypnotics or a combination of these agents) and individual or group psychotherapy,
(4) age 18–65 years,
(5) existence of legal capacity.

The exclusion criteria were:

(1) use of any psychoactive substance (including cannabis) during life, except alcohol, caffeine and nicotine,
(2) comorbid severe mental disorders (comorbid severe depressive disorder, bipolar affective disorder and/or psychosis),
(3) treatment for AUD not mentioned above,
(4) severe somatic disorders, including malignancy, hematological disease and untreated chronic somatic conditions,
(5) malnutrition (BMI < 18.5 kg/m^2^) or severe obesity (BMI > 35 kg/m^2^),
(6) pregnancy and breastfeeding,
(7) severe symptoms of alcohol withdrawal (scores > 7 on the Clinical Institute Withdrawal Assessment for Alcohol Scale, Revised (CIWA-Ar) (25),
(8) impairment of hearing and/or vision,
(9) intellectual disabilities or dementia,
(10) self-reported acute infection in the last month or/and an inflammatory systemic disease or autoimmune disease,
(11) previously diagnosed hyperlipidemia treated with antihyperlipidemic drugs,
(12) liver disease of non-alcoholic etiology, diagnosis of acute alcohol hepatitis, and liver transplantation and
(13) patient withdrawal from research.

These inclusion and exclusion criteria also applied to the assessment at the second time point. The only exception was that some participants were unable to maintain abstinence, so relapse was included as a moderator in our analyses.

The participants were divided into two groups. They were classified as belonging to the COVID-19 (exposed) group if they reported at least one COVID-19–related symptom and subsequently had a confirmed positive PCR test. Individuals were assigned to the non-COVID (non-exposed) group if they self-reported no history of COVID-19–related symptoms and had never tested positive on either a PCR or antigen test. The medical data of participants were also reviewed. Participants with PCR or antigen-confirmed asymptomatic infections were excluded, as our classification required concordance between symptoms and test results. Thus, classification as exposed required the presence of both a positive PCR test and COVID-19–related symptoms, whereas classification as non-exposed required the absence of both. Participants assigned to the non-exposed group who reported COVID-19 in the last six months at the second assessment were excluded from statistical analyses.

The possibility of exposure classification bias could not be avoided because asymptomatic cases or unreported mild symptoms in the non-exposed group cannot be completely ruled out, especially given the long time period and the variability of COVID-19 presentations (26–28). Nevertheless, the applied strict inclusion and exclusion criteria allowed us to examine whether there are differences between individuals with symptoms clearly attributable to COVID-19 and those without such symptoms.

### 2.3 Study measures

1. Socio-demographic data (age, gender, educational level, working status, marital and housing status, parenthood, number of children, and socioeconomic status).
2. Clinical interview about recovery from symptomatic COVID-19 disease (number of episodes, time elapsed since the last positive SARS-CoV-2 test, treatment characteristics, vaccination status). COVID-19-related symptoms were defined as any symptoms that temporally and clinically correlated with the presence of confirmed SARS-CoV-2 infection, including exacerbations of pre-existing conditions. The list of symptoms is comprehensive and detailed because SARS-CoV-2 infection can affect any organ system (26–28).
3. The Alcohol Use Disorders Identification Test (AUDIT) uses 10 questions to self-assess the severity of alcohol use in the last year. Scores of 8 or higher are interpreted as hazardous or harmful alcohol use (29). Cronbach's alpha, as a measure of reliability, in our sample was α_1_ (first assessment) = 0.90 and α_2_ (second assessment) = 0.91.
4. The Penn Alcohol Craving Scale (PACS) is a self-reported measure of alcohol craving over the past week (30). This scale measures the frequency, intensity, duration of craving, resistance to alcohol use and overall craving. Items are scored from 0 to 6, for a maximum of 30 points. Scores of 15 or above show clinically significant craving (30, 31). Cronbach's alpha in our sample was α_1_ (first assessment) = 0.91 and α_2_ (second assessment) = 0.93.
5. The Montreal Assessment of Cognitive Functions (MoCA) is a screening instrument for detecting cognitive impairment (32), which is widely used in research involving AUD individuals (33). The MoCA is a brief, 30-point assessment that is usually completed in 10 min, and a cutoff score of 26 is typically used, with scores of 25 or below indicating impairment (32). Kuder-Richardson 20 in our sample was KR-20 (first assessment) = 0.74 and KR-20 (second assessment) = 0.70.
6. Inflammatory (ESR, CRP), biochemical (AST, ALT, GGT) and lipid (TG, TC, LDL, HDL) blood parameters were determined using automated analyzers. Typical reference values used in everyday clinical practice in Croatia were applied: ESR (5–28 mm/h), CRP (up to 5 mg/L), AST (8–30 U/L), ALT (10–36 U/L), GGT (9–35 U/L), TG (up to 1.7 mmol/L), TC (up to 5 mmol/L), LDL (up to 3 mmol/L), HDL (from 1.2 mmol/L). By measuring ESR and CRP, we aimed to assess potential immune upregulation; levels of liver enzymes were examined to evaluate possible hepatic dysfunction; and lipid parameters were analyzed to identify possible metabolic disturbances.

The same sociodemographic questionnaire, COVID-19 interview, measurement instruments and blood parameters were assessed at two time points.

### 2.4 Data analysis

Descriptive statistics were calculated for all sociodemographic variables of AUD patients in the COVID-19 and non-COVID-19 groups. To assess distribution normality, we examined skewness and kurtosis for alcohol use, alcohol craving, cognitive functions, inflammatory, biochemical and lipid parameters within both groups and conducted the Shapiro-Wilks test.

The goal of this paper is to explore differences in alcohol use, alcohol craving, cognitive functions, inflammatory, biochemical and lipid parameters between AUD patients who were exposed to symptomatic COVID-19 disease, and those who were not, in two, six-months separated time points. However, two important variables emerged during the design of analytical strategy. First, the relapse in alcohol use is possible related to all dependent variables of interest (9, 13, 34, 35), and there were 52 % of patients who relapsed between two measurement time points (58 % in non-COVID-19 group, and 44 % in COVID-19 group). Relapse status was determined through a clinical interview and the AUDIT. Relapse was defined as the consumption of at least one standard drink on at least one occasion within the previous six months before the second assessment. It was introduced as a binary variable in our analyses. Second, in the COVID-19 group, patients were unbalanced in the time passed since the last positive PCR test. Moreover, the time passed from the last positive PCR test also varied within a patient between the first and second measurement, making it a time-varying covariate. To address all the aspects of the dataset (between and within participant factors and their interactions, and time-varying covariates), data analysis was conducted by linear mixed-effects modeling. In all models, random intercepts were defined at the level of participant to model the repeated measurements within a participant. Alcohol use, alcohol craving, cognitive functions, inflammatory, biochemical and lipid parameters were dependent variables (DVs) in models. For each DV a two-model strategy was applied. In the first model the measurement time point (T1 vs. T2), COVID-19 group (Exposed to COVID-19 vs. not exposed to COVID-19) and relapse status (yes vs. no) were entered as fixed effects, and we looked at the main effects, all two-way and three-way interactions between them. The highest order interaction that was statistically significant was interpreted via Bonferroni corrected *post-hoc* analysis, and model-based estimated marginal means. This model was followed by the second one in which time since the positive PCR test (centered around its mean, where the value of 0 indicates the average time) was entered as a covariate. However, this was possible to conduct only within the COVID-19 subsample, and we inspected if the observed statistical effects remained the same after including the time since exposure as covariate.

We did not include additional covariates (sociodemographic data, vaccination status, COVID-19 disease severity and duration) in the model in order to preserve statistical power and avoid overfitting, due to the sample size.

Follow-up data are missing for five participants who discontinued AUD treatment and did not attend the second assessment.

Finally, to examine associations between variables within each group, we calculated Pearson's correlation coefficients, while also statistically controlling for relapse as a covariate. Effect sizes for Pearson's correlation coefficients were interpreted according to Cohen's criteria: small (±0.1), medium (±0.3), and large (±0.5) (36). Multiple comparison correction was not applied in the correlational analysis, and this analysis is exploratory in nature.

Participants with missing values for a specific variable (due to non-compliance) were excluded from analyses involving that variable only.

All analyses were conducted in Jamovi v.2.6 (37) software.

## 3 Results

### 3.1 Descriptive statistics and normality test

One participant was excluded from the analyses, since they were not exposed to the COVID-19 at the first study point, but had a positive PCR test before the second time point, thus not having clear membership to none of study groups.

Majority of 63 AUD patients were men, secondary educated, employed, married or in a relationship, having children, living with partner and children, and having an average socioeconomic status. 51.7 % of participants experienced an alcohol relapse after 6 months.

Descriptive statistics indicated that the groups were comparable across most sociodemographic and clinical characteristics, although some differences were observed in age, sex distribution, educational level, and the frequency of alcohol relapse (Table 1). The COVID-19 group was slightly younger than the non-COVID-19 group, with a relatively small difference in mean age and substantial overlap in age distributions between the groups (48.03 ± 9.3 vs. 52.2 ± 8.73 years). Women were more frequently represented in the COVID-19 group compared with the non-COVID-19 group (40.6 % vs. 22.6 %), whereas men predominated in the non-COVID-19 group (77.4 %). Participants in the COVID-19 group more often had tertiary education (34.4 % vs. 16.2 %), while secondary education was more common in the non-COVID-19 group (67.7 % vs. 46.9 %). The alcohol relapse was more frequent in the non-COVID-19 group than in the COVID-19 group (58.1 % vs. 44.4 %). Employment rates, housing status, parental status, number of children, marital status, and socioeconomic status were generally similar between the two groups.

**Table 1:** Sociodemographic and COVID-19 disease characteristics of alcohol use disorder patients [COVID-19 (*N* =32) and non-COVID-19 group (*N* = 31)] (*N* = 63).

Variable	Levels	Overall sample	COVID-19 group	Non-COVID-19 group	*p*
Age (mean ± SD), years		50.10 ± 9.04	48.03 ± 9.31	52.23 ± 8.73	0.065
Gender	Women	20 (31.7)	13 (40.6)	7 (22.6)	0.124
	Men	43 (68.3)	19 (59.4)	24 (77.4)	
Educational level^*^	Primary	8 (12.7)	5 (15.6)	3 (9.7)	0.254
	Secondary	36 (57.1)	15 (46.9)	21 (67.7)	
	Tertiary education level	16 (25.4)	11 (34.4)	5 (16.2)	
	Master's degree or doctorate	3 (4.8)	1 (3.1)	2 (6.5)	
Working status	Unemployed	12(19.0)	7 (21.9)	5 (16.1)	0.493
	Employed	42 (66.7)	22 (68.7)	20 (64.6)	
	Retired	9 (14.3)	3 (9.4)	6 (19.4)	
Marital status	Single	10 (15.9)	5 (15.6)	5 (16.1)	0.904
	Married or in a relationship	34 (54)	17 (53.2)	17 (54.8)	
	Divorced	16 (25.4)	9 (28.1)	7 (22.6)	
	Widowed	3 (4.8)	1 (3.1)	2 (6.5)	
Parents	Yes	48 (76.2)	25 (78.2)	23 (74.2)	0.714
	No	15 (23.8)	7 (21.8)	8 (25.8)	
Number of children (mean ± SD)		1.38 ± 1.02	1.28 ± 0.88	1.48 ± 1.51	0.436
Housing status	Alone	12 (19)	6 (18.8)	6 (19.4)	0.874
	With primary family	13 (20.6)	7 (21.9)	6 (19.4)	
	With child/children	6 (9.5)	3 (9.3)	3 (9.7)	
	With partner	13 (20.6)	8 (25.0)	5 (16.1)	
	With partner and child/children	19 (30.2)	8 (25.0)	11 (35.5)	
Socioeconomic status^**^	Below average	14 (22.2)	7 (21.9)	7 (22.6)	0.422
	Average	39 (61.9)	20 (62.5)	19 (61.3)	
	Above average	10 (15.9)	5 (15.6)	5 (16.1)	
Relapse	Yes	30 (51.7)	12 (44.4)	18 (58.1)	0.3
	No	28 (48.3)	15 (55.6)	13 (41.9)	
COVID-19 disease	Yes	32 (50.8)			
	No	31 (49.2)			
Received vaccine	Yes	40 (63.5)	22 (68.7)	18 (58.1)	0.378
	No	23 (36.5)	10 (31.3)	13 (41.9)	
Treatment due to COVID-19 disease	Outside the hospital	30 (93.7)			
	Hospital treatment	2 (6.3)			
Number of PCR-confirmed SARS-CoV-2 infections (mean ± SD)		1.31 ± 0.59			
Time since the last positive PCR test (mean ± SD), days		577.53 ± 430.21			
Number of vaccine doses received (mean ± SD)		2.41 ± 0.60	2.44 ± 0.71	2.38 ± 0.49	0.744
Time since the last vaccine dose (mean ± SD), months		19.55 ± 5.80	16.88 ± 4.61	21.71 ± 5.86	0.009

SD, standard deviation, *p* – chi square or *t*-test associated *p*-value.

^*^ Years of education = primary (8 years), secondary (12 years), tertiary education level ( ≤ 15 years), master's degree or doctorate (>15 years).

^**^based on self-reported individual and family income.

Regarding COVID-19 status, 51 % of participants had contracted symptomatic COVID-19 disease. The average number of symptomatic SARS-CoV-2 infections was 1.31 ± 0.59. The average time since the last positive PCR test was 577.53 ± 430.21 days, and the average number of vaccine doses received was 2.41 ± 0.60. The time since the last vaccine dose was 19.55 ± 5.80 months. Among those who experienced symptomatic COVID-19 disease, only 6.3 % required hospitalization. Overall, 63.5 % of participants were vaccinated, with a slightly higher vaccination rate in the COVID-19 group compared with the non-COVID-19 group (68.7 % vs. 58.1 %) (Table 1).

Furthermore, we additionally tested if there are significant differences between the COVID-19 and non-COVID-19 groups in the aforementioned descriptive variables. It has been shown that there are no differences in age, number of children and number of vaccine doses, and proportion of genders, education levels, working statuses, marital statuses, having children, housing status, socioeconomic statuses, and relapse in these two groups of patients (all *p* > 0.05, Table 1). The only observed statistically significant difference was regarding the time of last vaccination, where the COVID-19 group (16.88 ± 4.61) had significantly shorter time since last vaccine compared to the non-COVID-19 group (21.71 ± 5.86).

The results of Shapiro-Wilks test (Supplementary Table 1) indicated that most of variables differ statistically significant from the theoretically normal distribution. However, these deviations were deemed mild and unlikely to substantially affect the results of the linear mixed effects models. First, the group size is equal, and there are repeated measurements within participant, increasing the total number of observations. Second, skewness and kurtosis coefficients fell within acceptable limits: most values fall below the thresholds of 3 for skewness and 10 for kurtosis (38).

### 3.2 Linear mixed-effects models: group, time, relapse and their interactions

Results of linear mixed-effects model for testing differences in alcohol use, alcohol craving, cognitive functions, inflammatory, biochemical and lipid parameters between AUD patients who were exposed to symptomatic COVID-19 disease, and those who were not, in two, six-months separated time points are presented in Table 2, while the results of *post-hoc* comparisons and estimated marginal means for the statistically significant results are presented in the Supplementary Table 2. The analysis indicated that there were no statistically significant differences in ESR, CRP, GGT, TG, and LDL regarding the time, group, relapse fixed effects and their interaction terms. Only the time main effect was statistically significant for HDL, indicating an average increase in this parameter in T2 for all patients. PACS, AST, and ALT showed higher values on average present among patients who relapsed in comparison to those who did not. A more complex results pattern was observed with the MoCA scores, where the time^*^group, and time^*^relapse two-way interaction terms were statistically significant. The interpretation of *post-hoc* tests, and estimated marginal means revealed that the MoCA scores increased from T1 to T2 but only for participants in the non-COVID-19 group, while the scores remained the same for COVID-19 patients. Furthermore, the MoCA scores increased from T1 to T2, among patients who did not relapse, while they remained the same for patients who relapsed between T1 and T2. Lastly, the time^*^group^*^relapse three-way interaction was statistically significant for the TC parameter. It has been shown that TC decreased from T1 to T2 in COVID-19 patients who relapsed, and increased in COVID-19 patients who did not relapse.

**Table 2:** Results of linear mixed effects models for testing the effects of time, and alcohol relapse within COVID-19 and non-COVID-19 alcohol use disorder patients on alcohol use severity and craving, cognitive functions, inflammatory, biochemical and lipid parameters.

DV	Model parameters		Fixed effects	*F*	Interpretation
AUDIT	N_obs_	121	T	*F*_(1, 55.768)_ = 42.167, *p* = < 0.001	Decreased in non-relapse group, remained the same in relapse group
	N_part_	63	G	*F*_(1, 57.809)_ = 1.38, *p* = 0.245	
	C R^2^	0.603	R	*F*_(1, 57.809)_ = 23.671, *p* = < 0.001	
	M R^2^	0.349	T^*^G	*F*_(1, 55.768)_ = 0.004, *p* = 0.949	
	σ^2^ int	30.803	T^*^R	*F*_(1, 55.768)_ = 10.73, *p* = 0.002	
	σ^2^ res	48.029	G^*^R	*F*_(1, 57.809)_ = 0.053, *p* = 0.818	
	ICC	0.391	T^*^G^*^R	*F*_(1, 55.768)_ = 0, *p* = 0.991	
PACS	N_obs_	121	T	*F*_(1, 57.825)_ = 2.127, *p* = 0.15	Higher in relapse group, compared to non-relapse group
	N_part_	63	G	*F*_(1, 59.074)_ = 0.893, *p* = 0.348	
	C R^2^	0.352	R	*F*_(1, 59.074)_ = 15.456, *p* = < 0.001	
	M R^2^	0.149	T^*^G	*F*_(1, 57.825)_ = 0.011, *p* = 0.916	
	σ^2^ int	6.922	T^*^R	*F*_(1, 57.825)_ = 0.995, *p* = 0.323	
	σ^2^ res	22.122	G^*^R	*F*_(1, 59.074)_ = 0.039, *p* = 0.844	
	ICC	0.238	T^*^G^*^R	*F*_(1, 57.825)_ = 0.085, *p* = 0.772	
MOCA	N_obs_	121	T	*F*_(1, 56.157)_ = 10.754, *p* = 0.002	Increased in non-COVID-19 group Increased in non-relapse group
	N_part_	63	G	*F*_(1, 59.129)_ = 0.334, *p* = 0.565	
	C R^2^	0.621	R	*F*_(1, 59.129)_ = 0.005, *p* = 0.944	
	M R^2^	0.108	T^*^G	*F*_(1, 56.157)_ = 6.875, *p* = 0.011	
	σ^2^ int	7.643	T^*^R	*F*_(1, 56.157)_ = 6.879, *p* = 0.011	
	σ^2^ res	5.661	G^*^R	*F*_(1, 59.129)_ = 1.662, *p* = 0.202	
	ICC	0.574	T^*^G^*^R	*F*_(1, 56.157)_ = 2.555, *p* = 0.116	
ESR	N_obs_	100	T	*F*_(1, 45.053)_ = 3.991, *p* = 0.052	No statistically significant effects
	N_part_	59	G	*F*_(1, 54.4)_ = 2.269, *p* = 0.138	
	C R^2^	0.557	R	*F*_(1, 54.4)_ = 2.801, *p* = 0.1	
	M R^2^	0.095	T^*^G	*F*_(1, 45.053)_ = 0.463, *p* = 0.5	
	σ^2^ int	52.452	T^*^R	*F*_(1, 45.053)_ = 2.715, *p* = 0.106	
	σ^2^ res	50.323	G^*^R	*F*_(1, 54.4)_ = 0.19, *p* = 0.665	
	ICC	0.51	T^*^G^*^R	*F*_(1, 45.053)_ = 0.28, *p* = 0.599	
CRP	N_obs_	105	T	*F*_(1, 44.7)_ = 0.797, *p* = 0.377	No statistically significant effects
	N_part_	60	G	*F*_(1, 56.323)_ = 0.339, *p* = 0.563	
	C R^2^	0.749	R	*F*_(1, 56.323)_ = 0.029, *p* = 0.866	
	M R^2^	0.014	T^*^G	*F*_(1, 44.7)_ = 0.91, *p* = 0.345	
	σ^2^ int	734.28	T^*^R	*F*_(1, 44.7)_ = 0.052, *p* = 0.821	
	σ^2^ res	251.297	G^*^R	*F*_(1, 56.323)_ = 0.006, *p* = 0.941	
	ICC	0.745	T^*^G^*^R	*F*_(1, 44.7)_ = 0.8, *p* = 0.376	
AST	N_obs_	115	T	*F*_(1, 49.314)_ = 0.382, *p* = 0.539	Higher in relapse group
	N_part_	62	G	*F*_(1, 51.526)_ = 0.436, *p* = 0.512	
	C R^2^	0.271	R	*F*_(1, 51.526)_ = 6.433, *p* = 0.014	
	M R^2^	0.082	T^*^G	*F*_(1, 49.314)_ = 0, *p* = 0.989	
	σ^2^ int	152.604	T^*^R	*F*_(1, 49.314)_ = 0.056, *p* = 0.814	
	σ^2^ res	586.573	G^*^R	*F*_(1, 51.526)_ = 2.105, *p* = 0.153	
	ICC	0.206	T^*^G^*^R	*F*_(1, 49.314)_ = 0.027, *p* = 0.87	
ALT	N_obs_	114	T	*F*_(1, 52.094)_ = 2.1, *p* = 0.153	Higher in relapse group
	N_part_	62	G	*F*_(1, 56.248)_ = 0.384, *p* = 0.538	
	C R^2^	0.401	R	*F*_(1, 56.248)_ = 4.595, *p* = 0.036	
	M R^2^	0.081	T^*^G	*F*_(1, 52.094)_ = 0.855, *p* = 0.359	
	σ^2^ int	152.342	T^*^R	*F*_(1, 52.094)_ = 0.274, *p* = 0.603	
	σ^2^ res	285.013	G^*^R	*F*_(1, 56.248)_ = 1.577, *p* = 0.214	
	ICC	0.348	T^*^G^*^R	*F*_(1, 52.094)_ = 0.076, *p* = 0.783	
GGT	N_obs_	116	T	*F*_(1, 58.118)_ = 0.767, *p* = 0.385	No statistically significant effects
	N_part_	62	G	*F*_(1, 60.406)_ = 1.947, *p* = 0.168	
	C R^2^	0.324	R	*F*_(1, 60.406)_ = 3.979, *p* = 0.051	
	M R^2^	0.096	T^*^G	*F*_(1, 58.118)_ = 1.907, *p* = 0.173	
	σ^2^ int	24,101.83	T^*^R	*F*_(1, 58.118)_ = 1.669, *p* = 0.202	
	σ^2^ res	71,396.81	G^*^R	*F*_(1, 60.406)_ = 2.934, *p* = 0.092	
	ICC	0.252	T^*^G^*^R	*F*_(1, 58.118)_ = 1.81, *p* = 0.184	
TG	N_obs_	113	T	*F*_(1, 39.556)_ = 0.42, *p* = 0.52	No statistically significant effects
	N_part_	62	G	*F*_(1, 46.234)_ = 1.864, *p* = 0.179	
	C R^2^	0.579	R	*F*_(1, 46.234)_ = 0.641, *p* = 0.427	
	M R^2^	0.086	T^*^G	*F*_(1, 39.556)_ = 0.216, *p* = 0.645	
	σ^2^ int	6,571.499	T^*^R	*F*_(1, 39.556)_ = 1.63, *p* = 0.209	
	σ^2^ res	5,612.564	G^*^R	*F*_(1, 46.234)_ = 3.676, *p* = 0.061	
	ICC	0.539	T^*^G^*^R	*F*_(1, 39.556)_ = 1.314, *p* = 0.259	
TC	N_obs_	113	T	*F*_(1, 49.997)_ = 7.712, *p* = 0.008	For patients that relapsed - decrease of TC in COVID-19 group For patients that did not relapse - increase of TC in COVID-19 group
	N_part_	62	G	*F*_(1, 56.015)_ = 5.341, *p* = 0.025	
	C R^2^	0.582	R	*F*_(1, 56.015)_ = 0.671, *p* = 0.416	
	M R^2^	0.223	T^*^G	*F*_(1, 49.997)_ = 5.835, *p* = 0.019	
	σ^2^ int	143.357	T^*^R	*F*_(1, 49.997)_ = 17.309, *p* = < 0.001	
	σ^2^ res	166.864	G^*^R	*F*_(1, 56.015)_ = 0.338, *p* = 0.563	
	ICC	0.462	T^*^G^*^R	*F*_(1, 49.997)_ = 18.234, *p* = < 0.001	
LDL	N_obs_	112	T	*F*_(1, 58.448)_ = 0.174, *p* = 0.678	No statistically significant effects
	N_part_	61	G	*F*_(1, 59.197)_ = 0.167, *p* = 0.684	
	C R^2^	0.095	R	*F*_(1, 59.197)_ = 0.983, *p* = 0.326	
	M R^2^	0.033	T^*^G	*F*_(1, 58.448)_ = 0.072, *p* = 0.79	
	σ^2^ int	70.297	T^*^R	*F*_(1, 58.448)_ = 0.469, *p* = 0.496	
	σ^2^ res	1,035.392	G^*^R	*F*_(1, 59.197)_ = 0.359, *p* = 0.551	
	ICC	0.064	T^*^G^*^R	*F*_(1, 58.448)_ = 0.868, *p* = 0.355	
HDL	N_obs_	113	T	*F*_(1, 58.036)_ = 4.604, *p* = 0.036	Increase in T2
	N_part_	62	G	*F*_(1, 60.178)_ = 0.218, *p* = 0.643	
	C R^2^	0.244	R	*F*_(1, 60.178)_ = 0.566, *p* = 0.455	
	M R^2^	0.1	T^*^G	*F*_(1, 58.036)_ = 2.137, *p* = 0.149	
	σ^2^ int	7.499	T^*^R	*F*_(1, 58.036)_ = 0.306, *p* = 0.582	
	σ^2^ res	39.215	G^*^R	*F*_(1, 60.178)_ = 3.917, *p* = 0.052	
	ICC	0.161	T^*^G^*^R	*F*_(1, 58.036)_ = 0.179, *p* = 0.674	

DV, dependent variable; Nobs, Number of observations; Npart, Number of participants; C R^2^, Conditional R^2^; M R^2^, Marginal R^2^; σ^2^ int, random intercept variance; σ^2^ res, residual variance; ICC, intraclass correlation coefficient; T, time; G, group; R, relapse; AUDIT, Alcohol Use Disorders Identification Test; PACS, Penn Alcohol Craving Scale; MoCA, Montreal Cognitive Assessment; ESR, erythrocyte sedimentation rate; CRP, c-reactive protein; AST, aspartate aminotransferase; ALT, alanine aminotransferase; GGT, gamma-glutamyl transferase; TG, triglycerides; TC, total cholesterol; LDL, low-density lipoproteins; HDL, high-density lipoproteins.

Linear mixed effects models where the fixed effects of time and relapse were controlled for time-varying covariate time since the positive PCR test are presented in the Supplementary Table 3, while accompanying *post-hoc* analyses and estimated marginal means are presented in the Supplementary Table 4. It has been shown that including time since the last positive PCR test as a covariate does not impact the interpretation of the previously presented main and interaction effects of time, group and relapse on alcohol use, alcohol craving, cognitive functions, inflammatory, biochemical and lipid parameters between AUD patients who were exposed to symptomatic COVID-19 disease, and those who were not, in two, six-months separated time points. This analysis shows that time since PCR is in low and negative relationship with CRP (*b* = −0.025, *p* = 0.009).

### 3.3 Pearson correlation analysis: associations between variables in COVID-19 and non-COVID-19 group

In the COVID-19 group (Table 3), large and positive correlations were observed between: AST and ALT, and AST and GGT. Furthermore, medium and positive correlations were observed between next pairs of variables: AUDIT and PACS, MOCA and TC, AST and CRP, GGT and ALT, and TC and TG. Furthermore, medium and negative correlations were observed among: HDL with ESR, AST, ALT, and GGT.

**Table 3:** Pearson correlation coefficients: associations between alcohol use severity, alcohol craving, cognitive functions, inflammatory, biochemical and lipid parameters in COVID-19 (*N* = 32, below the diagonal) and non-COVID-19 (*N* = 31, above the diagonal) alcohol use disorder patients.

Variables	AUDIT	PACS	MoCA	ESR	CRP	AST	ALT	GGT	TG	TC	LDL	HDL
AUDIT	—	0.343^**^	−0.397^**^	0.128	−0.11	−0.124	−0.078	0.03	−0.101	−0.152	−0.013	−0.149
PACS	0.45^***^	—	−0.198	0.455^**^	0.062	−0.017	0.007	0.065	−0.03	−0.134	0.03	−0.082
MoCA	0.042	−0.111	—	−0.106	0.013	−0.092	−0.064	−0.134	−0.019	0.144	−0.042	0.181
ESR	0.211	−0.092	−0.03	—	0.205	0.046	0.155	0.34^*^	0.012	0.139	0.005	−0.059
CRP	0.014	0.052	−0.182	0.269	—	0.399^**^	0.368^**^	0.047	0.229	0.207	−0.042	−0.099
AST	−0.036	0.125	−0.123	0.039	0.308^*^	—	0.876^***^	0.511^***^	−0.077	−0.171	−0.172	−0.019
ALT	−0.057	0.117	−0.23	−0.037	0.263	0.877^***^	—	0.509^***^	−0.139	−0.107	−0.133	−0.072
GGT	−0.028	0.1	−0.037	0.035	−0.068	0.517^***^	0.425^**^	—	0.04	−0.057	−0.052	−0.047
TG	−0.011	0.11	0.061	−0.054	0.017	−0.125	0.018	−0.082	—	0.292^*^	−0.104	−0.118
TC	−0.051	0.147	0.297^*^	0.106	−0.157	−0.118	−0.055	−0.226	0.309^*^	—	0.315^*^	0.332^*^
LDL	−0.068	0.067	−0.05	0.025	−0.11	−0.019	0.062	0.08	0.138	0.255	—	0.441^***^
HDL	0.075	0.107	0.109	−0.305^*^	−0.177	−0.292^*^	−0.449^**^	−0.29^*^	−0.088	−0.013	−0.096	—

AUDIT, Alcohol Use Disorders Identification Test; PACS, Penn Alcohol Craving Scale; MoCA, Montreal Cognitive Assessment; ESR, erythrocyte sedimentation rate; CRP, c-reactive protein; AST, aspartate aminotransferase; ALT, alanine aminotransferase; GGT, gamma-glutamyl transferase; TG, triglycerides; TC, total cholesterol; LDL, low-density lipoproteins; HDL, high-density lipoproteins;

^*^p < 0.05,

^**^p < 0.01,

^***^p < 0.001.

In the non-COVID-19 group (Table 3), large and positive correlations were observed among ALT and AST, AST and GGT and ALT and GGT. Furthermore, medium and positive correlations were observed among pairs of variables: AUDIT and PACS, PACS and ESR, ESR and GGT, CRP and AST, CRP and ALT, TG and TC, TC and LDL, TC and HDL, and finally HDL and LDL. On the other hand, negative and medium correlation was observed between AUDIT and MOCA scores.

## 4 Discussion

In this pilot study we examined the initial differences between COVID-19 and non-COVID-19 AUD patients in alcohol use, craving, cognitive functions, and selected blood parameters (inflammatory, biochemical, lipid) in two separated time points, as well as correlations among variables within each group.

Firstly, our results show that the most of our COVID-19 AUD patients experienced mild COVID-19 disease. Furthermore, our main findings indicate that COVID-19 and non-COVID-19 group differ in cognitive functions, while no significant differences were observed in AUD severity and blood parameters.

Cognitive functions improved after six months only in the non-COVID group. Previous research show that COVID-19 disease may result in impairments across all cognitive domains, which persist for more than 12 months (39). Cognitive symptoms are more often reported in cases of severe COVID-19, in unvaccinated individuals and those with symptomatic COVID-19 disease earlier in the pandemic period (40). However, COVID-19 in non-hospitalized individuals may also be associated with certain long-term cognitive impairments, due to central inflammatory processes and neuronal injury even in milder cases (40–42). AUD patients with COVID-19 history should undergo regular cognitive screening, since cognitive functions play an essential role in successful AUD treatment and AUD patients are already vulnerable to cognitive impairment (9, 43).

Our main findings further suggest that AUD patients with a history of mild COVID-19 disease do not significantly differ in AUD severity, and inflammatory, biochemical and lipid parameters from AUD patients without a history of COVID-19. However, this null finding should be interpreted with caution. Future multi-center studies should include larger samples to enable control of other covariates and the detection of smaller between-group effects. Also, further research is needed to assess different outcomes in relation to the time elapsed since recovery. CRP levels decrease as more time passes since positive PCR testing, implying that the inflammatory response associated with mild COVID-19 disease in AUD patients could attenuate over time. Inflammatory markers are primarily elevated in cases of acute and severe forms of COVID-19 disease due to more severe systemic inflammatory response syndrome (SIRS) (44), which may explain the absence of significant differences in blood parameters between the two groups. Moreover, it is surprising that the most of our COVID-19 AUD patients experienced mild COVID-19, because alcohol use is associated with numerous consequences related to SIRS, and impairment in both cellular and humoral immunity (3, 45, 46). However, it is possible that our participants did not experience severe COVID-19 disease because they do not suffer from severe somatic comorbidities, are all younger than 65 years, mostly vaccinated, and more than 40 % of them are women (26–28, 47).

Further findings from this study can be divided into three distinct groups: (a) relapse-related differences (b) time changes possibly related to reduction in alcohol use (c) differences in the associations between variables.

As expected, participants who relapsed have increased AUD severity and higher alcohol craving, as well as higher levels of liver enzymes (mainly ALT and AST) in comparison to non-relapse group. Furthermore, non-relapse participants show improvement in cognitive functions after six months, in contrast to the relapse group. Reduction in alcohol use has beneficial effects on all aspects of cognitive functioning. Abstinence promotes brain neuroplasticity and enables the recovery of cognitive functions to a partial or even full extent in some individuals (48). Most cognitive functions recover within 6 to 12 months after the establishment of initial abstinence, whereas some cognitive functions, such as verbal functions, already improve within the first month (49).

The lipid profile, specifically TC, shows a distinct pattern of change in COVID-19 patients depending on relapse status. Alcohol adversely affects fatty acid metabolism in the liver, resulting in disturbances in lipid levels in the blood (50). However, since studies in the general population have shown that alcohol cessation may worsen the cholesterol profile, which is also suggested by our findings, long-term research in the abstinent-AUD population is needed (51). Also, dyslipidemia in this group could be associated with COVID-19 (12). SARS-CoV-2 specifically targets lipid-secreting cells and uses the lipid metabolism of host cells for virus replication and systemic spreading, thus disrupting cholesterol homeostasis (12, 52). Furthermore, since recovered SARS-CoV-1 patients exhibit altered lipid levels even 12 years after the initial infection, we suggest long-term monitoring of lipid parameters for AUD patients with a COVID-19 history (12, 53).

The increasing levels of HDL after six months in all participants suggests benefits of reduced alcohol use and AUD treatment (13). However, the significant improvement observed after six months may also reflect the natural course of lipid improvement in AUD and a potential regression-to-the-mean effect. Therefore, further studies including a control group without AUD treatment are needed to confirm any causal relationship.

Some associations between variables are only observed in the non-COVID-19 group. There is a positive association between alcohol craving and ESR in this group. Patients with AUD who develop delirium tremens have the highest circulating ESR levels (54, 55). Research shows that inflammatory markers can be used as indicators of treatment response and disease severity in AUD, further confirming inflammatory etiology of AUD (46). Furthermore, higher AUD severity was associated with poorer cognitive functioning in the non-COVID-19 group, which is consistent with previously discussed absence of cognitive improvement after six months in the relapse group. Vice versa, individuals with better cognitive functioning achieve more favorable treatment outcomes (43). Unexpectedly, higher TC levels were associated with better cognitive functioning in the COVID-19 group, further suggesting possible impairments in lipid levels related to COVID-19, as previously discussed (13, 52, 53). However, this correlational analysis is exploratory in nature, and future studies are needed to explore the causal relationship between these variables.

Finally, the COVID-19 group included a higher number of women and participants with completed tertiary education compared with the non-COVID-19 group. Previous research show that women are more likely to self-report prior infection (56–58), and they have, as well as individuals with higher education, better COVID-19–related health literacy, which is positively associated with adherence to COVID-19 measures (59–61). Furthermore, the significant difference in the time elapsed since the last vaccination between groups is not surprising, as individuals with a history of COVID-19 were recommended to receive the vaccine after a certain period after recovery (62, 63).

### 4.1 Strenghts and limitations

The strength of this study is that the eligibility of participants was evaluated by an addiction subspecialist using detailed diagnostic criteria. The aim was addressed comprehensively using validated measurement instruments. Furthermore, we included patients who fulfilled strict eligibility criteria, thus limiting the potential impact of numerous confounders on the outcomes.

This study lacks pretesting conducted prior to the COVID-19 pandemic, so we cannot measure the direct effect of COVID-19 disease on the outcomes. Although we did a follow-up study, a longer follow-up is needed. Future studies with a larger sample size should account for additional covariates, such as sociodemographic variables, vaccination, COVID-19 disease severity and duration, other factors (pattern and amount of alcohol use, diet, physical activity). Our methodology reduced the risk of misattributing symptoms to other conditions and did not selectively include only severe COVID-19 cases. However, future studies should include serological testing to reduce possibility of classification bias (e.g., including individuals with unrecognized asymptomatic or mild infection in the non-exposed group). Future studies should also consider selection (e.g., hospital-based recruitment, predominance of male participants) and recall (regarding COVID-19 history) bias. For these limitations, the results of this study are not generalizable to the broader population of AUD patients, but should instead be viewed as a basis for future research in this area.

However, this study offers an initial insight into the relationship between symptomatic COVID-19 disease and both clinical and blood parameters in AUD patients. This study also adds important evidence from Croatia and underrepresented region, a context in which empirical data on AUD and post-COVID-19 recovery are limited.

## 5 Conclusions

Our findings suggest that cognitive functions, inflammatory and lipid parameters require long-term follow-up in COVID-19 AUD patients. Improvements in clinical and blood parameters were observed in the non-relapse group, which supports the overall benefits of AUD treatment and reduction of alcohol use.

## Data Availability

The raw data supporting the conclusions of this article will be made available by the authors, without undue reservation.
