# Supplementary material for: Initial insights into the relationship between symptomatic COVID-19 disease and specific clinical and blood parameters in patients with alcohol use disorder
Source: Front Public Health. 2026 Apr 23;14:1773558. doi: 10.3389/fpubh.2026.1773558 (PMC13149245; doi:10.3389/fpubh.2026.1773558)
Supplement: Supplementary file 1 [file Data_Sheet_1.pdf]

Supplementary materials

Supplementary Table 1. Skewness, kurtosis and Shapiro-Wilks test of normality of alcohol use severity, alcohol craving, cognitive functions, inflammatory, biochemical and lipid parameters in COVID-19 (N = 32) and non-COVID-19 (N = 31) alcohol use disorder patients (N =63).

	COVID-19 group				Non-COVID-19 group			
	Skewness	*Kurtosis*	*S-W*	*df*	*Skewness*	*Kurtosis*	*S-W*	*df*
AUDIT	-0.48	-0.63	0.92***	25	-0.26	-1.07	0.915***	23
PACS	1.08	-0.32	0.718***	25	1.4	0.8	0.719***	23
MoCA	0.13	-0.44	0.921***	25	0.41	-1.04	0.97	23
ESR	1.85	4.12	0.792***	25	1.38	2.9	0.869***	23
CRP	1.71	2.89	0.725***	25	3.78	15.62	0.802***	23
AST	3.13	8.99	0.453***	25	3.21	10.46	0.673***	23
ALT	1.92	3.48	0.688***	25	2.6	7.65	0.763***	23
GGT	2.04	4.47	0.237***	25	4.68	22.23	0.392***	23
TG	1.03	0.24	0.714***	25	0.66	-0.68	0.932**	23
TC	0.33	1.02	0.888***	25	0.14	-0.86	0.947*	23
LDL	-0.01	0,25	0.961	25	0.68	0.63	0.337***	23
HDL	0,51	-0,03	0.945*	25	0.84	-0.42	0.936**	23

Note. S-W = Shapiro-Wilks statistic; AUDIT = Alcohol Use Disorders Identification Test; PACS = Penn Alcohol Craving Scale; MoCA = Montreal Cognitive Assessment; ESR = erythrocyte sedimentation rate; CRP = c-reactive protein; AST = aspartate aminotransferase; ALT = alanine aminotransferase; GGT = gamma-glutamyl transferase; TG = Triglycerides; TC= total cholesterol; LDL = low-density lipoproteins; HDL = high-density lipoproteins; $^{***}p < 0.001$;$^{**}p < 0.05$ $^{*}p < 0.01$. Alcohol use severity = AUDIT, alcohol craving = PACS, cognitive functions = MoCA, inflammatory = ESR, CRP, biochemical = AST, ALT, GGT and lipid parameters = TG, TC, LDL, HDL.

Supplementary Table 2. Post hoc comparisons and estimated marginal means for the linear mixed effects models for testing the effects of time, and alcohol relapse within COVID-19 and non-COVID-19 alcohol use disorder patients on alcohol use severity and craving, cognitive functions, inflammatory, biochemical and lipid parameters.

AUDIT	Post hoc test T*R interacion									
	T	Relapse	vs	T	Relapse	Difference	SE	t	df	pbonferroni
	T1	No	-	T1	Yes	-5.147	2.289	-2.249	98.647	0.161
	T1	No	-	T2	No	12.607	1.824	6.913	57.241	< .001
	T1	No	-	T2	Yes	-0.994	2.289	-0.434	98.647	1
	T1	Yes	-	T2	No	17.754	2.349	7.559	101.219	< .001
	T1	Yes	-	T2	Yes	4.153	1.826	2.274	54.337	0.162
	T2	No	-	T2	Yes	-13.601	2.349	-5.791	101.219	< .001
	Estimate Marginal Means - T ✻ Relapse					95% Confidence Intervals				
	T	Relapse	Mean	SE	df	Lower	Upper			
	T1	No	15.312	1.582	98.647	12.173	18.45			
	T1	Yes	20.458	1.654	98.647	17.175	23.741			
	T2	No	2.704	1.667	103.583	-0.602	6.01			
	T2	Yes	16.306	1.654	98.647	13.023	19.588			
PACS	Post hoc test Relapse main effect									
	Relapse	vs	Relapse	Difference	SE	t	df	pbonferroni		
	No	-	Yes	-4.349	1.106	-3.931	59.074	< .001		
	Estimate Marginal Means - Relapse				95% Confidence Intervals					
	Relapse	Mean	SE	df	Lower	Upper				
	No	1.644	0.774	60.866	0.096	3.192				
	Yes	5.993	0.79	57.403	4.411	7.575				
MOCA	Post hoc test T*G interaction									
	T	Group	vs	T	Group	Difference	SE	t	df	pbonferroni

T1	Covid	-	T1	Non Covid	1.65	0.94	1.755	86.796	0.496
T1	Covid	-	T2	Covid	-0.292	0.644	-0.454	57.184	1
T1	Covid	-	T2	Non Covid	-0.971	0.94	-1.033	86.796	1
T1	Non Covid	-	T2	Covid	-1.942	0.96	-2.024	90.176	0.276
T1	Non Covid	-	T2	Non Covid	-2.622	0.612	-4.281	55.043	< .001
T2	Covid	-	T2	Non Covid	-0.679	0.96	-0.708	90.176	1

Estimate Marginal Means - T ✻ Group					95% Confidence Intervals	
T	Group	Mean	SE	df	Lower	Upper
T1	Covid	23.467	0.666	86.796	22.143	24.79
T1	Non Covid	21.816	0.664	86.796	20.497	23.136
T2	Covid	23.759	0.693	93.222	22.382	25.135
T2	Non Covid	24.438	0.664	86.796	23.119	25.757

Post hoc test T*R interacion

T	Relapse	vs	T	Relapse	Difference	SE	t	df	pbonferroni
T1	No	-	T1	Yes	-1.106	0.94	-1.176	86.796	1
T1	No	-	T2	No	-2.622	0.63	-4.165	57.283	< .001
T1	No	-	T2	Yes	-1.398	0.94	-1.486	86.796	0.845
T1	Yes	-	T2	No	-1.516	0.96	-1.58	90.176	0.706
T1	Yes	-	T2	Yes	-0.292	0.627	-0.465	55.043	1
T2	No	-	T2	Yes	1.225	0.96	1.276	90.176	1

Estimate Marginal Means - T ✻ Group					95% Confidence Intervals	
T	Group	Mean	SE	df	Lower	Upper
T1	No	22.088	0.65	86.796	20.797	23.38

	T1	Yes	23.194	0.68	86.796	21.843	24.545					
	T2	No	24.711	0.678	93.514	23.365	26.056					
	T2	Yes	23.486	0.68	86.796	22.135	24.837					
AST	Post hoc test Relapse main effect											
	Relapse	vs	Relapse	Difference	SE	t	df	pbonferroni				
	No	-	Yes	-14.407	5.68	-2.536	51.526	0.014				
	Estimate Marginal Means - Relapse				95% Confidence Intervals							
	Relapse	Mean	SE	df	Lower	Upper						
	No	23.616	3.944	51.457	15.699	31.532						
	Yes	38.023	4.088	51.591	29.818	46.227						
ALT	Post hoc test Relapse main effect											
	Relapse	vs	Relapse	Difference	SE	t	df	pbonferroni				
	No	-	Yes	-9.868	4.603	-2.144	56.248	0.036				
	Estimate Marginal Means - Relapse				95% Confidence Intervals							
	Relapse	Mean	SE	df	Lower	Upper						
	No	22.226	3.207	56.688	15.802	28.649						
	Yes	32.094	3.302	55.836	25.479	38.71						
TC	Post hoc test T*G*R interaction											
	T	Group	Relapse	vs	T	Group	Relapse	Difference	SE	t	df	pbonferroni
	T1	Covid	No	-	T1	Covid	Yes	-22.57	6.495	-3.475	87.454	0.022
	T1	Covid	No	-	T1	Non Covid	No	-11.714	6.603	-1.774	92.38	1
	T1	Covid	No	-	T1	Non Covid	Yes	-17.07	5.793	-2.947	87.454	0.115
	T1	Covid	No	-	T2	Covid	No	-8.301	4.706	-1.764	51.742	1
	T1	Covid	No	-	T2	Covid	Yes	12.355	6.997	1.766	95.827	1
	T1	Covid	No	-	T2	Non Covid	No	-10.506	6.34	-1.657	87.454	1
	T1	Covid	No	-	T2	Non Covid	Yes	-16.424	5.862	-2.802	88.956	0.175

T1	Covid	Yes	-	T1	Non Covid	No	10.856	7.289	1.489	91.529	1
T1	Covid	Yes	-	T1	Non Covid	Yes	5.5	6.564	0.838	87.454	1
T1	Covid	Yes	-	T2	Covid	No	14.269	6.838	2.087	93.554	1
T1	Covid	Yes	-	T2	Covid	Yes	34.926	5.881	5.939	51.409	< .001
T1	Covid	Yes	-	T2	Non Covid	No	12.064	7.051	1.711	87.454	1
T1	Covid	Yes	-	T2	Non Covid	Yes	6.146	6.624	0.928	88.632	1
T1	Non Covid	No	-	T1	Non Covid	Yes	-5.356	6.672	-0.803	92.284	1
T1	Non Covid	No	-	T2	Covid	No	3.413	6.942	0.492	97.274	1
T1	Non Covid	No	-	T2	Covid	Yes	24.07	7.74	3.11	97.482	0.069
T1	Non Covid	No	-	T2	Non Covid	No	1.208	5.393	0.224	49.11	1
T1	Non Covid	No	-	T2	Non Covid	Yes	-4.71	6.731	-0.7	93.252	1
T1	Non Covid	Yes	-	T2	Covid	No	8.769	6.176	1.42	94.822	1
T1	Non Covid	Yes	-	T2	Covid	Yes	29.426	7.061	4.167	95.69	0.002
T1	Non Covid	Yes	-	T2	Non Covid	No	6.564	6.411	1.024	87.454	1
T1	Non Covid	Yes	-	T2	Non Covid	Yes	0.646	4.397	0.147	46.989	1
T2	Covid	No	-	T2	Covid	Yes	20.656	7.317	2.823	99.481	0.161
T2	Covid	No	-	T2	Non Covid	No	-2.205	6.692	-0.33	93.807	1

T2	Covid	No	-	T2	Non Covid	Yes	-8.123	6.241	-1.302	95.816	1
T2	Covid	Yes	-	T2	Non Covid	No	-22.861	7.516	-3.042	94.808	0.085
T2	Covid	Yes	-	T2	Non Covid	Yes	-28.78	7.118	-4.043	96.42	0.003
T2	Non Covid	No	-	T2	Non Covid	Yes	-5.918	6.473	-0.914	88.688	1

Estimate Marginal Means - T ✻ Group ✻ Relapse						95% Confidence Intervals	
T	Group	Relapse	Mean	SE	df	Lower	Upper
T1	Covid	No	34.263	4.041	87.454	26.232	42.294
T1	Covid	Yes	56.833	5.084	87.454	46.728	66.938
T1	Non Covid	No	45.977	5.223	95.095	35.609	56.345
T1	Non Covid	Yes	51.333	4.151	87.454	43.083	59.584
T2	Covid	No	42.564	4.573	99.79	33.491	51.637
T2	Covid	Yes	21.908	5.712	99.278	10.574	33.242
T2	Non Covid	No	44.769	4.885	87.454	35.061	54.478
T2	Non Covid	Yes	50.687	4.246	90.288	42.252	59.123

HDL	Post hoc test Time main effect						
	T	vs	T	Difference	SE	t	df	pbonferroni
	T1	-	T2	-2.626	1.224	-2.146	58.036	0.036

Estimate Marginal Means - T				95% Confidence Intervals	
T	Mean	SE	df	Lower	Upper
T1	11.723	0.907	103.358	9.923	13.523
T2	14.349	0.962	104.101	12.441	16.256

Note. SE- standard error; t- t-test results; df- degrees of freedom; pbonferroni- Bonferonni corrected p-value.

Supplementary Table 3. Results of linear mixed effects models for testing the effects of time, and alcohol relapse within COVID-19 alcohol use disorder patients, while controlling for time since positive PCR test, on alcohol use severity and craving, cognitive functions, inflammatory, biochemical and lipid parameters.

	Model Parameters		Fixed effects	F	Interpretation
AUDIT	N_{obs}	59	T	F(1, 27.045) = 24.453, p = < .001	Decreased in no relapse group, remained the same in relapse group
	N_{part}	32	R	F(1, 28.915) = 9.185, p = 0.005	
	C R^2	0.677	Time since PCR	F(1, 53.104) = 0.716, p = 0.401	
	M R^2	0.34	T*R	F(1, 26.056) = 4.712, p = 0.039	
	σ^2 int	41.844			
	σ^2 res	40.199			
	ICC	0.51			
PACS	N_{obs}	59	T	F(1, 29.469) = 0.789, p = 0.382	Higher in relapse group, compared to non-relapse group
	N_{part}	32	R	F(1, 29.311) = 8.538, p = 0.007	
	C R^2	0.149	Time since PCR	F(1, 51.643) = 0.167, p = 0.684	
	M R^2	0.141	T*R	F(1, 29.455) = 0.084, p = 0.773	
	σ^2 int	0.335			
	σ^2 res	32.914			
	ICC	0.01			
MOCA	N_{obs}	59	T	F(1, 27.39) = 0.106, p = 0.747	Increased in non-relapse group
	N_{part}	32	R	F(1, 29.584) = 0.84, p = 0.367	
	C R^2	0.613	Time since PCR	F(1, 51.925) = 0.8, p = 0.375	
	M R^2	0.083	T*R	F(1, 26.321) = 8.816, p = 0.006	
	σ^2 int	8.123			
	σ^2 res	5.933			
	ICC	0.578			
ESR	N_{obs}	51	T	F(1, 21.852) = 1.174, p = 0.29	No statistically significant effects
	N_{part}	30	R	F(1, 27.531) = 2.165, p = 0.152	
	C R^2	0.72	Time since PCR	F(1, 41.337) = 0.249, p = 0.62	
	M R^2	0.114	T*R	F(1, 21.781) = 4.258, p = 0.051	

	σ^2 int	67.562			
	σ^2 res	31.238			
	ICC	0.684			
CRP	N_{obs}	51	T	$F(1, 23.432) = 0.28, p = 0.602$	
	N_{part}	30	R	$F(1, 29.418) = 0.08, p = 0.779$	
	C R^2	0.709	Time since PCR	$F(1, 38.212) = 7.473, p = 0.009$	
	M R^2	0.104	T*R	$F(1, 22.282) = 1.094, p = 0.307$	Time since PCR in low and negative relationship with CRP
	σ^2 int	583.666			
	σ^2 res	281.018			
	ICC	0.675			
AST	N_{obs}	54	T	$F(1, 21.072) = 0.147, p = 0.706$	
	N_{part}	31	R	$F(1, 22.604) = 5.971, p = 0.023$	
	C R^2	0.269	Time since PCR	$F(1, 48.561) = 0.001, p = 0.97$	
	M R^2	0.119	T*R	$F(1, 20.37) = 0.002, p = 0.965$	Higher in relapse group
	σ^2 int	160.36			
	σ^2 res	776.29			
	ICC	0.171			
ALT	N_{obs}	53	T	$F(1, 23.511) = 1.854, p = 0.186$	
	N_{part}	31	R	$F(1, 24.692) = 6.623, p = 0.016$	
	C R^2	0.171	Time since PCR	$F(1, 47.859) = 0.03, p = 0.862$	
	M R^2	0.151	T*R	$F(1, 22.884) = 0.229, p = 0.637$	Higher in relapse group
	σ^2 int	10.65			
	σ^2 res	426.514			
	ICC	0.024			
GGT	N_{obs}	55	T	$F(1, 29.014) = 1.187, p = 0.285$	
	N_{part}	31	R	$F(1, 30.382) = 3.499, p = 0.071$	
	C R^2	0.292	Time since PCR	$F(1, 49.904) = 0.007, p = 0.932$	No statistically significant effects
	M R^2	0.095	T*R	$F(1, 28.164) = 1.595, p = 0.217$	
	σ^2 int	40080.68			

	σ^2 res	143816.5			
	ICC	0.218			
TG	N_{obs}	54	T	F(1, 18.658) = 0.419, p = 0.525	No statistically significant effects
	N_{part}	31	R	F(1, 25.111) = 2.627, p = 0.118	
	C R^2	0.811	Time since PCR	F(1, 33.724) = 0.013, p = 0.909	
	M R^2	0.093	T*R	F(1, 17.649) = 2.835, p = 0.11	
	σ^2 int	13764.12			
	σ^2 res	3624.74			
	ICC	0.792			
TC	N_{obs}	54	T	F(1, 24.114) = 7.094, p = 0.014	Decrease for patients who relapsed
	N_{part}	31	R	F(1, 27.015) = 0.013, p = 0.909	
	C R^2	0.513	Time since PCR	F(1, 48.959) = 1.368, p = 0.248	
	M R^2	0.263	T*R	F(1, 23.124) = 18.487, p = < .001	
	σ^2 int	139.6			
	σ^2 res	272.968			
	ICC	0.338			
LDL	N_{obs}	53	T	F(1, 25.336) = 0.062, p = 0.805	No statistically significant effects
	N_{part}	30	R	F(1, 26.375) = 0.477, p = 0.496	
	C R^2	0.16	Time since PCR	F(1, 47.533) = 0.47, p = 0.496	
	M R^2	0.029	T*R	F(1, 24.799) = 0.363, p = 0.552	
	σ^2 int	19.266			
	σ^2 res	123.519			
	ICC	0.135			
HDL	N_{obs}	54	T	F(1, 28.076) = 0.407, p = 0.529	No statistically significant effects
	N_{part}	31	R	F(1, 30.29) = 0.996, p = 0.326	
	C R^2	0.284	Time since PCR	F(1, 48.979) = 0.038, p = 0.847	
	M R^2	0.029	T*R	F(1, 27.215) = 0.004, p = 0.949	
	σ^2 int	8.433			
	σ^2 res	23.705			

ICC	0.262

Note. Nobs- Number of observations; Npart- Number of participants; C R^2- Conditional R^2; M R^2- Marginal R^2; σ^2 int- random intercept variance; σ^2 res- residual variance; ICC- intraclass correlation coefficient; T- time; G- group; R- relapse.

Supplementary Table 4. Post hoc analysis and estimated marginal means for linear mixed effects models for testing the effects of time, and alcohol relapse within COVID-19 alcohol use disorder patients, while controlling for time since positive PCR test, on alcohol use severity and craving, cognitive functions, inflammatory, biochemical and lipid parameters.

AUDIT	Post hoc test T*R interacion									
	T	Relapse	vs	T	Relapse	Difference	SE	t	df	pbonferroni
	T1	No	-	T1	Yes	-5.042	3.33	-1.514	44.379	0.822
	T1	No	-	T2	No	12.325	2.249	5.479	27.17	< .001
	T1	No	-	T2	Yes	-0.318	3.312	-0.096	44.163	1
	T1	Yes	-	T2	No	17.367	3.464	5.014	46.692	< .001
	T1	Yes	-	T2	Yes	4.724	2.648	1.784	26.092	0.517
	T2	No	-	T2	Yes	-12.643	3.474	-3.639	47.797	0.004
	Estimate Marginal Means - T ✻ Relapse					95% Confidence Intervals				
	T	Relapse	Mean	SE	df	Lower	Upper			
	T1	No	16.578	2.031	44.209	12.486	20.67			
	T1	Yes	21.62	2.626	44.278	16.329	26.911			
	T2	No	4.253	2.261	50.496	-0.288	8.794			
	T2	Yes	16.896	2.634	44.409	11.588	22.204			
PACS	Post hoc test Relapse									
	Relapse	vs	Relapse	Difference	SE	t	df	pbonferroni		
	No	-	Yes	-4.509	1.543	-2.922	29.311	0.007		
	Estimate Marginal Means - Relapse				95% Confidence Intervals					
	Relapse	Mean	SE	df	Lower	Upper				
	No	2.105	0.99	31.901	0.089	4.121				
	Yes	6.614	1.183	27.427	4.188	9.04				
MOCA	Post hoc test T*R interacion									
	T	Relapse	vs	T	Relapse	Difference	SE	t	df	pbonferroni
	T1	No	-	T1	Yes	-0.871	1.378	-0.632	42.677	1

	T1	No	-	T2	No	-2.217	0.867	-2.556	27.341	0.099
	T1	No	-	T2	Yes	0.913	1.371	0.666	42.428	1
	T1	Yes	-	T2	No	-1.345	1.428	-0.942	45.112	1
	T1	Yes	-	T2	Yes	1.784	1.019	1.75	26.487	0.55
	T2	No	-	T2	Yes	3.13	1.43	2.188	46.063	0.203
	Estimate Marginal Means - T ✻ Relapse					95% Confidence Intervals				
	T	Relapse	Mean	SE	df	Lower	Upper			
	T1	No	23.048	0.84	42.481	21.353	24.744			
	T1	Yes	23.92	1.087	42.56	21.728	26.111			
	T2	No	25.265	0.927	49.147	23.403	27.127			
	T2	Yes	22.135	1.09	42.711	19.937	24.333			
AST	Post hoc test Relapse									
	Relapse	vs	Relapse	Difference	SE	t	df	pbonferroni		
	No	-	Yes	-22.579	9.24	-2.444	22.604	0.023		
	Estimate Marginal Means - Relapse				95% Confidence Intervals					
	Relapse	Mean	SE	df	Lower	Upper				
	No	21.355	5.758	22.559	9.432	33.279				
	Yes	43.934	7.222	22.214	28.965	58.904				
ALT	Post hoc test Relapse									
	Relapse	vs	Relapse	Difference	SE	t	df	pbonferroni		
	No	-	Yes	-15.545	6.04	-2.574	24.692	0.016		
	Estimate Marginal Means - Relapse				95% Confidence Intervals					
	Relapse	Mean	SE	df	Lower	Upper				
	No	20.735	3.809	25.451	12.898	28.572				
	Yes	36.28	4.67	23.26	26.626	45.934				
TC	Post hoc test T*R interaction									
	T	Relapse	vs	T	Relapse	Difference	SE	t	df	pbonferroni
	T1	No	-	T1	Yes	-21.688	7.528	-2.881	44.571	0.036

T1	No	-	T2	No	-8.167	5.99	-1.364	22.855	1
T1	No	-	T2	Yes	12.021	8.163	1.473	47.109	0.885
T1	Yes	-	T2	No	13.521	7.938	1.703	46.236	0.571
T1	Yes	-	T2	Yes	33.709	7.585	4.444	24.087	0.001
T2	No	-	T2	Yes	20.188	8.627	2.34	48.515	0.141

Estimate Marginal Means - T ✻ Relapse					95% Confidence Intervals	
T	Relapse	Mean	SE	df	Lower	Upper
T1	No	34.622	4.67	44.521	25.214	44.031
T1	Yes	56.31	5.881	44.534	44.463	68.158
T2	No	42.789	5.351	48.338	32.033	53.546
T2	Yes	22.602	6.737	48.045	9.056	36.147

Note. SE- standard error; t- t-test results; df- degrees of freedom; pbonferroni- Bonferonni corrected p-value
